# Structural determinants of gender inequality: why they matter for adolescent girls’ sexual and reproductive health

**DOI:** 10.1136/bmj.l6985

**Published:** 2020-01-27

**Authors:** Asha S George, Avni Amin, Claudia Marques de Abreu Lopes, T K Sundari Ravindran

**Affiliations:** 1 School of Public Health, University of the Western Cape, Bellville, South Africa; 2 Department of Reproductive Health Research World Health Organization, Geneva, Switzerland; 3 United Nations University-International Institute for Global Health, Kuala Lumpur, Malaysia; 4 School of Public Health, University of Witwatersrand, Johannesburg, South Africa

## Abstract

More comprehensive understanding of gender inequality is required, particularly the broader structural drivers that underpin the political economy of gender power relations, say **Asha George and colleagues**

In sub-Saharan Africa, four out five new HIV infections among 15-19 year olds are in girls according to UNAIDS 2019 estimates.^1^ Surveys during 2011-16 showed that more than half of rural women aged 15–24 in sub-Saharan Africa had been pregnant before their 18th birthday,^2^ and as recently as 2016, 40% of young women in sub-Saharan Africa and 30% in South Asia were married while still children.^3^ These examples highlight how gender power relations profoundly affect adolescent girls with lifelong consequences.

Research on gender inequality in global health has focused on factors operating at the individual level (age of marriage, literacy, etc), household level (decision making, household composition^4^), or community level (social norms,^5^ ^6^ access to services^7^ ^8^). Although gender inequality is experienced by and between individuals, it is also a result of power relations that structure how societies are organised, laws are set, economies function, and ideologies are shaped.^9^ We review some of these structural determinants of gender inequality, unpacking what they are and why they matter, with a focus on the sexual and reproductive health of adolescent girls.

Puberty is a formative period of rapid physical, cognitive, social, emotional, and sexual development, when differences in gender roles and gender inequalities become ingrained.^10^ These influence adolescent mortality and risk factors everywhere but particularly in low income countries (tables 1 and 2). Gender norms that encourage men to be strong and take risks partly explain the health harming risk behaviours of boys.^11^ ^12^ Gender inequalities are also particularly harmful to the sexual and reproductive health of adolescent girls and reverberate with lifelong effects.

**Table 1:** Leading causes of adolescent deaths in low income countries by sex and age group, 2016 (WHO global health estimates)

Rank	10-14 year olds			15-19 year olds	
	Male	Female		Male	Female
1	Road injury	Malaria		Road injury	Maternal conditions
2	HIV/AIDS	HIV/AIDS		Interpersonal violence	Road injury
3	Malaria	Diarrhoeal diseases		HIV/AIDS	Diarrhoeal diseases
4	Diarrhoeal diseases	Road injury		Diarrhoeal diseases	HIV/AIDS
5	Meningitis	Lower respiratory infections		Tuberculosis	Meningitis

**Table 2:** Leading risk factors associated with adolescent deaths in low income countries by sex and age group, 2017*

Rank	10-14 year olds			15-19 year olds		
	Male	Female		Male	Female
1	Unsafe sex	Unsafe sex		Unsafe sex	Unsafe sex
2	Unsafe water	Unsafe water		Occupational risk	Unsafe water
3	Air pollution	Air pollution		Unsafe water	Child and maternal malnutrition
4	Child and maternal malnutrition	Child and maternal malnutrition		Alcohol use	Intimate partner violence
5	Impaired kidney function	Impaired kidney function		Impaired kidney function	Air pollution

^*^ Data from Global Burden of Disease studies (www.healthdata.org/gbd)

Adolescent girls are vulnerable to acquiring and being harmed by sexually transmitted infections because of both biological and social factors. Adolescent girls tend to receive less education and information about sexuality and reproduction and have poorer access to health services than boys. They are also at higher risk of unsafe sex, often in situations where they have less control over sexual and reproductive decision making. Moreover, the risk of unsafe sex is compounded by the high risks of intimate partner violence and sexual violence faced by adolescent girls.^13^ Adolescent girls are also specifically targeted by harmful practices such as child marriage and, in some regions, female genital mutilation. They are less likely to complete secondary school or have secure employment as they transition into adulthood, face a higher burden of household work, and have less decision making autonomy, including restricted mobility compared with their male peers.^14^ If the sustainable development goals are to be realised without leaving behind those most in need, tackling the gendered dynamics that shape adolescent health, and especially the sexual and reproductive rights of adolescent girls, is critical.

## Why structural determinants are important

Structural determinants are the socioeconomic and political processes that structure hierarchical power relations, stratifying societies based on class, occupational status, level of education, gender, etc.^15^ They shape the environments that facilitate or impede people’s ability to protect themselves from illness, and if sick, their access to quality healthcare. They mould the social contexts that affect people’s experience of being sick, their health outcomes, and the socioeconomic consequences of being ill.^16^ ^17^Altering these power relations that shape social environments and contexts inequitably is possible but requires a conscious focus on social justice. 

When implemented over time policies that tackle structural determinants can achieve long term population effects and reach wider coverage than those focused on household or community level action.^18^ Action on these structural factors is therefore necessary to maximise and sustain the effect of clinical and behavioural interventions.^17^

## How structural determinants influence health outcomes and inequities

**Fig 1:**
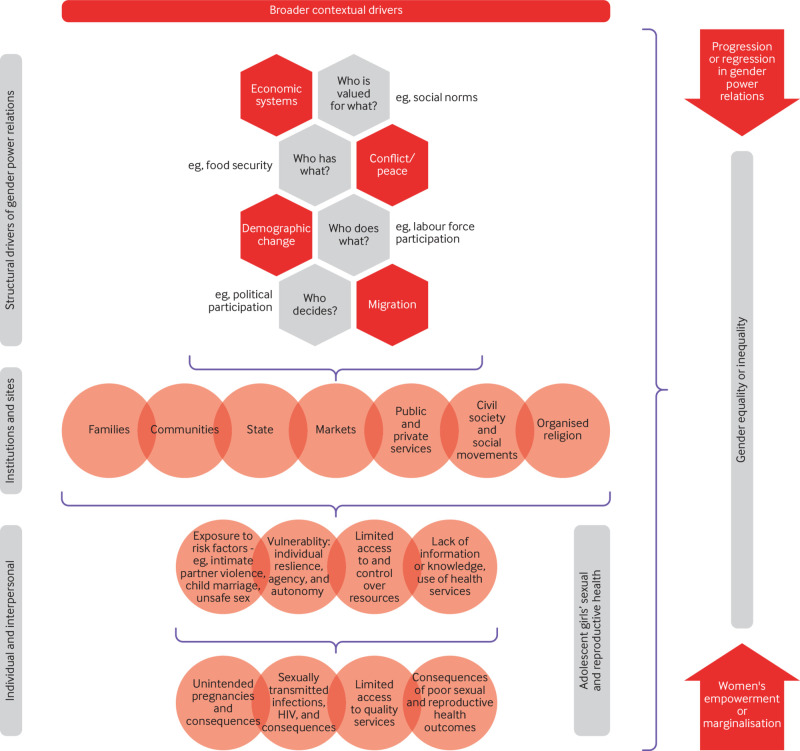
Conceptual framework for structural elements of gender power relations that drive gender inequality^18^ ^19^ ^20^

Building on earlier efforts,^15^ ^17^ we present a conceptual model that acknowledges a broad range of structural factors that interconnect to produce health inequity, such as economic systems, conflict and peace, migration, and other demographic transitions (fig 1). We cannot cover everything in the model within this article so focus on four main structural determinants that underpin gender power relations. These factors determine who has what (material and other assets), who does what (division of labour between market and reproductive labour), who decides (political participation and laws), and who is valued for what (social norms, ideologies).^19^ ^20^ They shape the institutions—including families, communities, and markets—that replicate gender inequality, which in turn influences health exposures, vulnerabilities, access to services, and outcomes. For each factor we describe how structural determinants underpin gender power relations and shape adolescent girls sexual and reproductive health.

## Who is valued and for what?

Unsafe sex, determined by gendered norms and other structural factors, is the leading risk factor for adolescent death (table 2). Gender norms govern what is valued and considered acceptable for men and women. In most societies, norms tend to value and privilege what is male over what is female, legitimising patriarchy and camouflaging its unfairness.

Research with young adolescents (10-14 year olds) across six cities around the world (Baltimore (US), Ghent (Belgium), Nairobi (Kenya), Ile Ife (Nigeria), Asyūṭ (Egypt), and Shanghai (China)) shows that puberty brings different expectations for boys and girls. Girls’ worlds are restricted (in appearance, dress, mobility, access to information) and boys’ worlds expand.^21^ In many societies, adolescent girls are expected to be virgins, represent family or clan honour, be submissive in their sexual and intimate relationships, and not have knowledge or information about sexuality or reproduction. Gender norms often stigmatise girls who seek contraceptives, become pregnant, or are sexually abused.^12^ Hence, gender norms are increasingly recognised as an important influence in shaping health, particularly adolescent sexual and reproductive health.^7^

For adolescent girls, changes in gender norms are influenced by positive role models in families, schools, and communities as well as access to media and information. Broader societal change related to economic, environmental, and demographic changes in society (eg, urbanisation, migration, conflicts, technology, economic opportunities) also have profound effects.^22^ For example, the decline in child marriage rates in South Asia was driven largely by growing economic and educational opportunities for girls.^23^ Similarly, across 80 countries, increasing female employment was independently associated with positive trends in gender norms and stereotypes, separate from regional trends, growth in gross domestic product (GDP), and the structure of production underlying GDP (agricultural, industrial, etc). In addition, differences by region, GDP growth, and GDP production structures also affected gender norms.^24^

Although it may be tempting to assume that gender norms progress towards promoting equality over time, this may not always be the case. In times of economic crisis and in regions where countries have changed economic systems, norms have become more inequitable in favour of men, as signalled by increasing agreement with the statement that men have more right to a job than women in the World Values surveys.^24^ Context is also crucial. For example, girls’ education is more strongly associated with reduced risk of partner violence in countries where partner violence is widespread than in those where it is not.^25^

## Who has what?

Given that malnutrition is among the top five risk factors for adolescent death (table 2), we examine food security as a key asset linked to other structural determinants of adolescent wellbeing. Food security is deeply gendered. Women are more likely than men to be affected by severe food insecurity in Asia, Africa, and Latin America, with the widest gap in Latin America.^26^ In situations of severe food insecurity, gender bias against girls often occurs in food allocation within households.^27^

Poor food security is linked to worse health behaviours and outcomes, particularly for adolescent girls. Studies from several settings (eg, Brazil, United States, and sub-Saharan Africa) highlight links between food insecurity and sexually transmitted infections, including reduced use of condoms, increased likelihood of engaging in transactional sex, and decreased likelihood of adhering to antiretroviral therapy for HIV.^28^ ^29^ Adolescents experiencing chronic food insecurity and undernutrition are at increased risk of poor mental health,^30^ which in turn influences risky sexual behaviour.^31^ Pregnancies in undernourished adolescents pose higher risk of obstetric complications and poor newborn outcomes.^32^

Many political, economic, and environmental factors contribute to the gendered effects of food insecurity. Discrimination in land, property, and inheritance laws, access to low cost credit, and cuts in government agricultural subsidies disproportionately affect women across low and middle income countries.^33^ Volatility of food prices also disproportionately affects women, who are often responsible for management of food within households. Additionally, marginalised women seeking to find or produce affordable quality food may further increase their workloads or even compromise their own food consumption to save food for other family members.^34^ ^35^

## Who does what?

The global labour force participation rate (a measure of the working age population in or looking for employment) is in long term decline, with the gap between men and women remaining stubbornly large. In 2018, the rate for women was 48.5%, 26.5 percentage points below that for men.^36^ Yet women’s economic participation is associated with lower fertility rates, better birth spacing, and delayed marriage.^36^ ^37^

Adolescence is when most people transition from education to work and begin participating in the labour force. Although this is often to alleviate household poverty and to support families under duress, such participation can also afford them the possibilities of greater mobility, networks, information, and financial independence and agency. However, even when parity in primary and secondary education is reached, it does not translate into equal participation in the labour force. For example, the proportion of people not in education, employment, or training in 2012-15 across 28 low and middle income countries was almost twice as high for female youth (30%) as for male youth (16%).^14^

Several barriers prevent the economic participation of adolescent girls and young women.^34^ Gender norms that support early marriage and pregnancy for young women perceive women as primarily responsible for household and care work; this often restricts their mobility and prevents them from completing their education and entering the labour force. Discriminatory policies and practices that exclude pregnant adolescent girls from schooling further compound their disadvantage. Their entry into the work force is also hampered by a lack of information and access to social networks to help with job searches and career opportunities; limited opportunities for training, including a lack of child care; and preference of employers in some sectors to hire only young men.

The net effect of female labour force participation depends on the social and economic context, and this is particularly true for adolescent girls and young women.^38^ Women may enjoy greater personal autonomy if they work in jobs outside family farms and enterprises. However, for some women, this may be at the cost of working in exploitative, dangerous, or stigmatised activities or in precarious employment without social protection or maternity benefits and at high risk of sexual harassment.^39^ Furthermore, increasing women’s participation in low level employment may perpetuate negative gender norms.

There are also unintended consequences that are context specific. Partner violence is less prevalent in countries with a high proportion of women in the formal work force. However, earning money increases a woman’s risk of partner violence in countries where few women are paid for their work.^25^ In other contexts, higher levels of female participation in the workforce can erode traditional masculine norms that previously ensured that men must provide for women and their children, leaving women to fend for their children and themselves.^22^

## Who decides?

Political participation is the most explicit manifestation of the distribution of power, where change is relatively recent and woefully insufficient. Women are a small minority in formal elected and appointed leadership positions across the world, with only a handful of countries beginning to reach parity.

While the right to vote and to be part of formal government processes through parliaments and cabinets is a critical aspect of gender equality and women’s empowerment, the aspect of female political participation that is often neglected is the role of feminist movements in bringing progressive change in public health agendas. A study of 70 countries over 40 years has shown that the presence of autonomous women’s movements has been more important than women in parliaments or leadership for passing progressive laws tackling violence against women.^40^ Social movements have also had a critical role in ensuring access to HIV treatment and in advocating for sexual and reproductive rights.^41^

The participation of adolescent girls and young women in policy processes, social movements, and planning of programmes related to their health is hampered by power dynamics related to both age and sex as well as other elements of their social position such as ethnicity or class. Their participation in sexual and reproductive health programmes is varied, ranging from limited engagement in peer education programmes to decision making and leadership in policy and programme development, through youth led activist organisations and networks (eg, advocates for youth or youth coalition for sexual and reproductive health).^42^

In understanding contemporary adolescent social movements, the role of social media in reshaping political participation of young people in demanding sexual and reproductive health rights may be particularly influential. Even though gender disparities in access to social media disadvantage females, social media has created new opportunities for young feminists to organise and mobilise through blogs, sharing of stories, and Twitter campaigns (eg, #BlackLivesMatter, #MeToo, #TimesUp). It is also critical to understand how the engagement of young women in feminist movements has changed what issues are of most relevance to them in relation to sexual and reproductive health. Younger feminist organising is more intersectional and more fluid in its understanding of gender justice and non-normative sexualities than older women’s movements. At the same time, women’s movements in several countries are facing backlash even though there is greater access to information and awareness about sexuality and reproduction.

## What next?

We have made the case for unpacking structural forms of gender power relations using the example of the sexual and reproductive health of adolescent girls. Research and policy must continue to tackle gender inequalities in health experienced by individuals, families, and communities, but a broader understanding of structural forms of gender inequality is needed to sustain change over time. Food security offers a good example. In addition to recognising gender biases related to food distribution within households, reducing food insecurity calls for a wide range of upstream policy changes that affect gender inequalities. These include food trade, use of land resources, agriculture investment, land ownership and access, and education.

Gender power relations affect both males and females, but structural forms of gender inequality starkly concentrate disadvantage against girls and women. Although substantial gains have been made across various health and education gender indicators, gender parity in political and economic participation remain distant goal that may take more than 100 years to reach.^43^ Changes in gender ideology, which is a foundation for gender inequality, remain actively contested.

Much of the language of “leaving no one behind,” calling for attention to marginalised groups that may be excluded from progress towards the SDGs, presumes that equality is a matter of addressing the lack of inclusion. Yet attention to structural forms of gender inequality shows that the terms of inclusion are critical. Adolescent girls and young women already participate in a system that is highly inequitable, contributing to their continued marginalisation. Girls and women need social change that ensures more secure and dignified livelihoods, not more inclusion into systems of discrimination. Greater inclusion of women in electoral systems, political parties, and social movements that are ideologically opposed to gender equality will not eliminate gender inequalities.

Finally, the four forms of structural gender inequality are interconnected. For example, gender norms are driven by material access to and control over resources, as well as to economic participation. And, conversely, adolescent girls’ and women’s economic participation is hampered by gender norms related to early marriage and child bearing and raising roles. This means that changing one aspect of gender inequality can have unintentional effects on other forms of gender inequality. These effects are unpredictable, sometimes amplifying progress, sometimes cancelling each other out, and sometimes regressing. These elements of transforming gender power relations require further consideration of complexity in research and policy, with its corresponding emphasis on contextual strategic analysis aided by conceptual frameworks, consultative sectoral engagement, and more considerate time frames to track both intended and unintended trajectories of social change.^44^

**Key messages**

- Reaching those most left behind by health interventions requires structural policy initiatives across multiple forms of marginalisation
- Women and girls are particularly discriminated against in economic and political arenas
- Marginalised girls and women are not left out of health and development, but the terms of their inclusion are marginalising
- Progress on structural determinants is nuanced and not necessarily linear, given unintended consequences and conservative gender backlash
- Policy change to address gender power relations in one area can be sidelined by lack of reform in other areas

## Notes

Contributors and sources: ASG and AA led the conceptualisation of the paper. ASG, AA, CMAL, and SR jointly developed the outline and wrote various sections of the paper. ASG led the drafting and finalisation of the paper. All authors read the final version and approved it.

Provenance and peer review: Commissioned; externally peer reviewed.

This article is part of a series proposed by Countdown to 2030 for Women’s, Children’s and Adolescents’ Health and the Partnership for Maternal, Newborn and Child Health (PMNCH) hosted by the World Health Organization and commissioned by *The BMJ*, which peer reviewed, edited, and made the decisions to publish these articles. Open access fees are funded by the Bill and Melinda Gates Foundation and PMNCH.
